# Breastfeeding and contraception counseling: a qualitative study

**DOI:** 10.1186/s12884-022-04451-2

**Published:** 2022-02-25

**Authors:** Marit Pearlman Shapiro, Karina Avila, Erika E. Levi

**Affiliations:** Division of Family Planning, Department of Obstetrics and Gynecology and Women’s Health, Albert Einstein College of Medicine, Bronx, NY USA

**Keywords:** Breastfeeding, Postpartum, Contraception counseling, Reproductive justice

## Abstract

**Background:**

The objectives of this qualitative study were to better understand women’s experiences regarding contraceptive choice, breastfeeding intentions and the relationship between the two. Women are routinely presented with counseling on breastfeeding and contraception throughout their prenatal and postpartum care, but little is published on patients’ own priorities, desires and experiences of this peripartum counseling. This article aims to address this gap in the literature.

**Methods:**

Semi-structured interviews were conducted with patients in the immediate postpartum period. The interview guide explored: 1) timing and content of contraceptive counseling; 2) breastfeeding goals and expectations; 3) reasons for contraceptive choices; and 4) recommendations for counseling. Interview transcripts were coded to identify themes and analyzed.

**Results:**

Twenty interviews were conducted. The participants were reflective of our patient population in the Bronx, with ninety percent using Medicaid for insurance and fifteen percent concerned about food security in the past month, well-validated questions reflective of poverty and socioeconomic status. Three themes emerged from the interviews: (1) using contraception was described as a selfish decision by the mother without benefit to the newborn; (2) women felt pressure to breastfeed and saw the inability to breastfeed as a personal failure; and (3) medical providers were viewed as more trustworthy when it came to information regarding breastfeeding as opposed to contraceptive options, where decisions relied on anecdotes from friends or family.

**Conclusions:**

Most decision-making regarding breastfeeding and contraception relied on the personal experiences of the participants and their friends and family. A clear need for support for women who are unable to breastfeed and education about the benefits of contraception for the newborn was identified.

## Introduction

Patients in the postpartum period face a myriad of decisions about caring for themselves and their infants, including their contraceptive options and breastfeeding choices. Postpartum guidelines from the World Health Organization (WHO) and the American College of Obstetricians and Gynecologists (ACOG) include medical recommendations for women to exclusively breastfeed their infants for 6 months, and to aim to avoid pregnancy for at least 18 months after delivery [1, 2]. To accomplish the medical recommendations for pregnancy spacing, most women will necessitate the use of a modern method of contraception. Well established best practices from these governing organizations include encouraging women to breastfeed and providing counseling on its benefits, and providing contraceptive counseling during prenatal and postpartum care.

The contraceptive method chosen in the postpartum period depends on individual patient characteristics, such as medical history, preference and whether the patient is breastfeeding [3]. According to the Center for Disease Control’s Medical Eligibility Criteria for Contraceptive Use (US MEC) all methods, except for combined hormonal contraceptives due to risk of venous thromboembolic events, are considered safe in the postpartum period [4]. ACOG recommends that patients have access to long-acting reversible contraception (LARC) in the postpartum period. Two methods of LARC contraception are available in the United States, the contraceptive implant which is placed in the arm and the intrauterine device (IUD). The IUD is available as either a non-hormonal copper device FDA-approved for 10 years or as a levonorgestrel device in various formulations, approved for up to 7 years [5]. LARC methods may be initiated immediately after pregnancy, such as with a post-placental IUD placement or contraceptive implant insertion prior to discharge from the hospital after a birth [6].

Hormonal methods containing progestins are generally considered acceptable for nursing mothers at any time in the postpartum period. Both the levonorgestrel and copper IUDs have been extensively studied in breastfeeding users and are shown not to effect milk supply or infant development [7–14]. Placement of the etonogestrel contraceptive implant in the early postpartum period (1–3 days) has been shown to be noninferior to standard insertion time (4–8 weeks) for time to lactogenesis stage II, breastfeeding rates and infant growth [7]. Depot medroxyprogesterone acetate (DMPA) can be given as an intramuscular injection and does not adversely affect milk supply or infant growth and development [4, 15–17]. There are conflicting clinical recommendations on early postpartum initiation of DMPA and breastfeeding outcomes, however a systematic review concluded that current evidence is too methodologically weak to show this [18]. There is always a possibility that for some women, exposure to hormonal birth control may impact milk supply.

The benefits of breastfeeding to mother, baby and society have been extensively researched and are well beyond the scope of this introduction. Briefly, breastfeeding protects against infections and obesity in the infant, breast cancer, likely ovarian cancer and type 2 diabetes in the mother, as well as improved birth spacing. One meta-analysis suggests that near universal breastfeeding could prevent 823,000 deaths annually in children under 5 years old and 20,000 annual deaths from breast cancer [19]. The Baby-Friendly Hospital Initiative is a worldwide initiative designed to increase breastfeeding initiation and support. In order to receive this designation, hospitals must rigorously demonstrate their commitment to promoting breastfeeding, including providing education, enabling initiation of breastfeeding within one hour after birth, and not accepting free formula. A systematic review has shown that the more Baby-Friendly interventions patients are exposed to, the more likely they are to have improved breastfeeding outcomes at time of discharge from the hospital [20].

Though research is available on the safety and efficacy of contraceptive methods while breastfeeding, little is known about the extent to which patients have access to these research findings. More insight is needed into the decision-making process regarding the use of postpartum contraception and if breastfeeding intentions play a role, and if the findings of the medical community translate into the decisions of new mothers. It is unclear if patients are routinely counseled by healthcare providers on the potential or theoretical effects of contraception on breastfeeding, and if it does occur, what this counseling entails. Further, there is little documentation of where women are receiving the material that informs these decisions outside of traditional healthcare models and the weight given by patients to each of these sources of information. There is an even greater paucity of data answering these questions for women living in an urban setting with limited resources, particularly among people of color. The objectives of this study were to describe postpartum patients’ experiences and perspectives regarding: 1) the counseling they received about postpartum contraception; 2) the counseling they received about breastfeeding; and 3) the relationship between breastfeeding and their plans for birth control.

## Methods

### Study setting

Montefiore Medical Center is an academic setting located in the Bronx, home to the poorest congressional district in the country. The hospital provides care for patients experiencing social and economic barriers that have the potential to prevent them from achieving the WHO and ACOG recommendations for birth spacing and breastfeeding. To combat these barriers, several initiatives have been successfully implemented in the past few years.

The women who give birth at Montefiore Medical Center are vulnerable to rapid repeat unintended pregnancy. Among women who attended a prenatal visit at Montefiore, more than one-third did not return for a postpartum visit, highlighting a barrier to contraceptive initiation in the postpartum period [21]. In April of 2014, New York State Medicaid began allowing hospitals to be reimbursed for LARC devices placed during an admission for delivery. Immediate postpartum LARC has been available to all patients since July 2014.

As immediate postpartum LARC was being initiated, Montefiore was simultaneously undergoing an evaluation to receive Baby-Friendly Hospital designation. 25% of all pregnant people in the Bronx intend to exclusively breastfeed, however a larger proportion of patients at Montefiore achieve breast milk feeding. In 2009 and 2010, 38.7% of babies born at Montefiore were exclusively breastfed during their hospital stay. New York City’s Department of Health most recently reports that now over 90% of infants delivering at both of our Labor and Delivery Units were fed breast milk.

### Recruitment and enrollment

This was a qualitative study that consisted of one-on-one, in-depth interviews with women who were one to three days postpartum. Semi-structured interviews were conducted by the primary investigator (MPS) between July and October 2017 at Weiler Hospital. Twenty interviews were conducted, at which point thematic saturation was reached. A purposive sampling strategy was used to ensure a diverse background of ideas and opinions. We aimed to include nulliparous and multiparous patients, adolescents and older women, and patients from a variety of ethnic and racial backgrounds. Participants were eligible for this study if they were 14 years of age or older, were on the Albert Einstein Weiler Hospital postpartum service, and spoke English. Participants provided written informed consent. Most interviews lasted between 30 and 42 min, though one interview lasted 11 min, and were conducted immediately after obtaining informed consent or scheduled for a time prior to the patient’s discharge. Participants were compensated $30 for their time. The study was approved by the Albert Einstein College of Medicine Institutional Review Board.

### Interview procedures

The interviews were conducted before the pandemic in the hospital rooms on the postpartum unit prior to the participants discharge from the hospital. Participants were interviewed alone or with family members present, as per patient preference. The interviewer was not involved in providing healthcare for any of the participants in the study, had no prior relationship with any of the participants and did not know the participants. It was made clear to all participants through the informed consent process, as approved by our Institutional Review Board, that choosing to participate or not in the study would not impact the healthcare they received. Demographic and background information was obtained from participants including questions about their obstetric history and previous contraceptive use. A chart abstraction was conducted to collect information about the participant’s labor and delivery course, mode of delivery, gestational age at delivery, and infant admission to the neonatal intensive care unit (NICU). Informed consent was obtained from the participants to access their medical records to perform the chart abstraction. All study documents including consent forms were approved by the IRB. Our interview guide explored our participants’ breastfeeding goals and expectations, reasons for contraceptive choices, and perceived relationship between breastfeeding and contraception. The interview guide was refined in an iterative process as new themes emerged. Interviews were recorded and transcribed by Wordsworth Typing and Transcription, a HIPAA-compliant transcription service approved by Montefiore, and verified by the authors. The transcripts were stored as electronic files and sent via an encrypted, password protected Box folder accessible only to the authors.

### Data analysis

Interviews were analyzed following all data collection. A common coding structure was agreed upon by all authors using clear, objective descriptions and examples. Interview transcripts were coded separately by two co-authors trained in qualitative analysis to identify themes using Dedoose software [22]. The codes were analyzed for inter-coder reliability and any differences in assigned codes were discussed and settled by consensus among the coders as themes were defined and finalized. If consensus could not be reached, a third, trained analyst was available to offer insight. The themes that emerged from the interviews exhibited both the original intent of the interview guide as well as data that surfaced during the discussions.

## Results

### Characteristics of the study participants

The participants were reflective of our patient population in terms of average age and breakdown of ethnic background. Ninety percent of the participants had Medicaid for insurance. Significantly, 15% of our participants responded that they were concerned about food security in the past month, a well-validated question reflective of poverty and socioeconomic status.

All of the participants received contraceptive counseling, including being offered postpartum LARC and 100% of participants received counseling about breastfeeding during their admission. 80% of the study participants had some counseling about postpartum contraception during their prenatal care, and all of the women interviewed had been offered a long-acting reversible form of contraception on admission to the labor and delivery unit by medical providers. All of the study participants had been offered a postplacental intrauterine device (IUD) at the time of admission, and 6 women had received postplacental IUDs. Notably, 35% of the participants were still undecided about using a contraceptive method postpartum at the time of the interview. All but one of the participants were planning to breastfeed, including 100% of the participants who were undecided about contraceptive method.

Additional sociodemographic characteristics of the study participants collected from chart abstraction are described in Table 1.

**Table 1:** Demographics of interview participants

Demographics	n (%)
Age, mean (range)	25.8 (19 – 41)
Body Mass Index, mean (range)	31.6 (26.1 – 43.5)
Race/Ethnicity	
Latinx	12 (60%)
Black	4 (20%)
White	2 (10%)
Asian	2 (10%)
Partner Status	
Single	3 (15%)
Living with partner	12 (60%)
Married	5 (25%)
Postpartum Birth Control Method	
Post-placental Mirena	4 (20%)
Post-placental Paragard	2 (10%)
Interval IUD	3 (15%)
Nexplanon	1 (5%)
Depo-Provera	1 (5%)
Pills	2 (10%)
Undecided	7 (35%)
Contraceptive history	
Previously used contraception (including condoms)	14
Never used contraception	7
Length of time intending to breastfeed	
Not planning to breastfeed	1 (5%)
6 months	16 (80%)
1 year	3 (15%)
Received prenatal care	20 (100%)
Discussed contraception with their prenatal care provider	16 (80%)

Three major themes, described below, emerged from the interviews.

#### Theme 1: contraception as a selfish decision

Many women expressed the importance of contraception as a means of avoiding a future pregnancy thus postponing the physical discomforts and pain associated with pregnancy and birth. These descriptions were particularly earnest in the immediate postpartum period. Contraception was not seen as a tool for birth spacing, but rather as a means of being able to engage in intercourse while avoiding pregnancy. The majority of women did not believe birth control was beneficial for their child. When asked if they would theoretically prioritize contraception or breastfeeding, almost all of the women felt breastfeeding was more important because it prioritized and directly benefited their children.

*Sometimes you gotta take risks for yourself for your child, so breastfeeding, yeah, I would do it over birth control…. ‘Cause breastfeeding is benefitting my child, not me. You know, birth control you’re just being selfish, thinking about yourself.*

Participant 2, a 24 year old multiparous patient who had previously breastfed for 3 months

*The baby is a priority. Sex isn’t really a priority. That could be avoided. You can’t really avoid feeding your baby, so.*

Participant 8, a first-time mother who wanted to breastfeed

Six other participants spoke about the potential downsides of breastfeeding they were willing to endure for the benefits to their infant, including engorgement, mastitis and nipple trauma.

*I had such a hard time. Like my nipples actually, they split open. Yeah, and they, oh, it was so sore. I almost gave up.*

Participant 5, who breastfed her first two children for 1 and 2 years respectively

Conversely, only one woman out of the 20 interviewed stated that birth control was beneficial for her baby.

*Because I’m controlling how many babies I have. So I can take good care of her. If I have so many children, I don’t think I’m going to be able to take good care of all of them […] I’m not going to have 10 kids…but I could buy formula. You know? Like it’s not the best thing, but it’s not the best thing to have so many kids that you can’t really give them the attention or whatever.*

Participant 13, a primiparous former labor floor nurse from Colombia

Other than this sole participant, no other women discussed the advantages of birth spacing or planning a pregnancy as beneficial to their existing child or children or to their families.

#### Theme 2: inability to breastfeed as a personal failure

The majority of participants described feeling immense pressure from their medical providers to breastfeed their newborn infants. They spoke about being “pushed” to breastfeed or denied formula when they requested it. When mothers were unable or unwilling to breastfeed, they experienced feelings of guilt and failure. They used terms like “failure as a mom,” “self-loathing,” and “less of a woman” when discussing their inability to breastfeed. Several participants spoke about their personal disappointment that they were not successful at breastfeeding.

*‘Cause I feel like I should be able to [breastfeed]. I see other people do it and why can’t I?[…] But it’s definitely tough when I see other people able to do it and I’m just like, it’s so easy for them to do it and I am taking so long.*

Participant 6, the mother of a premature baby in the neonatal intensive care unit

A few participants spoke about how these emotions were formalized and reinforced by their interactions with their medical providers.

*I will say it this way, it’s kind of pushed…I was asking for more formula for her, and they were like, well, we’re not really a formula hospital, we’re all breastfeeding. And I was like, oh, okay. Like kind of put you in a situation where it’s like well, if I could I would, so I wouldn’t be asking you for the formula if I could breastfeed. So that’s where a little guilt came in too, because it keeps bringing it back to the fact that I personally can’t.*

Participant 19, a first-time mother

One patient described how a pediatrician reinforced the idea that she was personally responsible for her older child’s poorer health outcomes. She spoke about the emotional stress she experienced hearing that her actions may have caused pain and illness for her son.

*My boy gets sick a lot from ear infections and the doctor says that it probably has to do with not fully breastfeeding him, like side effects from not breastfeeding him.”*

Participant 13

#### Theme 3: trusted sources for information

While women receive formal counseling on both breastfeeding and contraception options from healthcare providers, they choose to rely on information from medical professionals much more when it concerns breastfeeding. Women reported being told that breastfeeding was the healthiest option for their baby and often cited this as a reason for why they chose to breastfeed. There were several women who described breaking with their family’s recommendations to use formula when deciding how to feed their child, instead choosing to breastfeed after learning about the benefits for their newborn from a medical provider.

Eighteen participants related anecdotes from friends and family about personal experiences with contraception. About half of the participants had made their own contraceptive decisions based on their friends’ or family members’ personal experiences with a given method.

*Interviewer: Have you used any kind of birth control before?*

*Participant 12: No, because I was always scared of birth control, ‘cause everybody kept telling me their experiences with birth control, and I’m just like, ‘Oh no.’*

*I heard from my mother’s friend—she ended up getting an IUD and she had an infection and she had to go to the hospital to get it surgically removed, because it was so bad that her infection, I guess, messed with the contraception. It got stuck somewhere in her uterus and they had to like hurry up and, you know, like take it out so that I didn’t really want.*

Participant 4, a 19 year old multiparous patient considering a contraceptive implant

While women receive formal counseling from healthcare providers regarding both breastfeeding and birth control, medical providers were viewed as more trustworthy when it came to information regarding breastfeeding as opposed to contraceptive options. Women described making decisions about breastfeeding based on information they received from the medical institution, even if contradictory to the previous experiences of their friends or family. Women were less likely to believe the information provided in formal counseling about side effects and less likely to use this information in their decision-making about contraception.

Additionally, one woman described her counseling experience:

*Two different doctors asked what type of birth control I wanted to get on. And instead of it feeling like they were informing me, it felt like they were pushing me to be on it…. I work in the medical field. And to me, it almost seems like somebody purchased something and now they have to sell that product. It’s kind of like forcing it. I mean, it’s fine, but it’s kind of being too forceful.*

Participant 15, a 36-year-old Black woman

This woman spoke at length about the coercion she experienced to use a LARC method, identifying forceful counseling that she received from multiple providers on the labor floor. While other women did not describe their counseling experience as coercive, all of the women interviewed did receive counseling on LARC methods on admission to the labor floor.

## Conclusions

This qualitative study exploring postpartum decision-making in a socioeconomically disadvantaged population identified several factors that potentially impede women from meeting the WHO and ACOG recommendations for birth spacing and breastfeeding. Women have internalized that breastfeeding is best for their babies, however this in turn has led to intense feelings of shame and failure among women who are unable or unwilling to achieve the goal of exclusive breastfeeding. Most of the women had been extensively counseled on the health benefits of breast milk, whether or not they were planning on breastfeeding. Weiler Hospital was certified as Baby Friendly in June 2017, which could have caused women to receive strong messages about breastfeeding. The pressure from the medical establishment is more straightforward to use a form of postpartum contraception, however this is not supported by the experiences of the friends and family trusted by the participants. These interviews have highlighted that the medical system needs to find a way to provide breastfeeding support and education to women without leading to increased shame and guilt among new mothers.

Perhaps the most surprising finding of the study was that the participants did not connect breastfeeding and contraception counseling to each other at all, particularly given their distinctive reactions to the counseling. They did not perceive contraception use and birth spacing as having any medical benefit to themselves or their babies. Consistently, the participants expressed the idea that using birth control was a way for women to have sex for pleasure without getting pregnant, not as a method of ensuring better health outcomes for themselves, their newborn or future pregnancies. Further research is needed to investigate where this perspective originates, as well as the most effective methods of dispelling this belief and providing education about the benefits of using contraception for birth spacing.

This is particularly relevant in light of another finding of the study that illustrated that women, especially socioeconomically disadvantaged women of color, remain distrustful of medical professionals when it comes to contraception. This is especially apparent when it comes to methods that are not under a woman's control and require implantation by a medical provider. Studies have shown that women of color and low socioeconomic status are more likely than white women to be offered LARC. Our findings suggest the negative impact of provider bias on patient experiences, when discussions of contraception options can easily turn coercive. The medical establishment must find a balance between providing education and offering options, and pressuring patients to use LARCs.

One limitation of the study is that no study participants underwent postpartum sterilization. This possibly excludes a cohort of women who had the firmest beliefs about their reproductive decision-making. Participants were interviewed in the immediate postpartum period while still hospitalized, in the middle of their decision-making processes and putting into practice previously made decisions prior to their deliveries. Additionally, only English-speaking participants were included in the study, excluding an important postpartum population in the United States.

The strength of the study was inclusion of women across a broad spectrum of demographics. The participants chose to use a wide variety of types of postpartum contraception. We have included a robust procedural description and believe these results are recognizable to the people who share this experience, and to those who care for the patient populations similar to our study participants. We hope these findings are generalizable to some extent throughout the United States where many hospitals care for underserved communities, particularly as postpartum LARC provision and Baby Friendly certification become more widespread. We believe these findings fit into contexts outside of the study situation and hope that clinicians view the findings as meaningful and applicable in their own experiences. It is unclear if given this unique patient population, similar patterns would emerge in other populations.

The findings from this study reinforce that the medical establishment needs to find a way to reframe the conversation around birth spacing to focus on the benefits to the newborn and maternal health. Similarly, these interviews illustrate a need for the conversation about breastfeeding to be centered around the experience of the mother. These conversations between prenatal care providers and women, and the resulting decisions made about pregnancy spacing and breastfeeding, have long-lasting implications for the wellbeing of these families. Counseling strategies that provide a sense of pride and wellbeing, rather than shame and guilt, need to be studied and implemented to better support women in the postpartum period.

## Data Availability

The datasets generated and analyzed during the current study are not publicly available due to logistics of publicly storing interview transcripts, but are available from the corresponding author on reasonable request.

## Abbreviations

ACOG: American College of Obstetricians and Gynecologists
IUD: Intrauterine device
LARC: Long-acting reversal contraception
MEC: Medical Eligibility Criteria for Contraceptive Use
NICU: Neonatal intensive care unit
WHO: World Health Organization
